# Community Prevention of Young Adult Drinking and Associated Problems

**Published:** 2004

**Authors:** Harold D. Holder

**Affiliations:** Harold D. Holder, Ph.D., is a senior research scientist at the Prevention Research Center, Pacific Institute for Research and Evaluation, Berkeley, California

**Keywords:** young adults, underage drinking, heavy drinking, prevention of problematic AOD (alcohol and other drug) use, community-based prevention, environmental-level prevention, social policy prevention approach, regulatory prevention approach, alcoholic beverage distribution laws, minimum drinking age laws, drinking and driving, law enforcement, The Saving Lives Project, The Community Trials Project, Communities Mobilizing for Change on Alcohol (CMCA), Robert Wood Johnson Foundation’s Fighting Back program

## Abstract

This article briefly summarizes three evidence-based community intervention trials sponsored by the National Institute on Alcohol Abuse and Alcoholism (NIAAA). Designed to reduce alcohol use among youth and young adults, these trials demonstrate the potency of community interventions that can influence the price, availability, drinking context, and perceived risks of heavy drinking among young people. The effectiveness of comprehensive, research-based local prevention efforts is confirmed by research examining other programs to reduce alcohol sales to youth as well as the harm caused by alcohol use among youth and young adults, including alcohol-related traffic accidents and assaults. By restructuring the total alcohol environment in a way that can be self-sustaining, these interventions are more likely to be effective than one-time interventions.

Community action is essential to preventing problems associated with drinking alcohol, and especially those related to heavy alcohol use among youth and young adults. The rationale behind targeting communities instead of a subgroup of young people, such as those enrolled at a particular school, is compelling. Whether they are working, attending college, or in the military, young adults typically are part of a community. The means through which young people usually obtain alcohol—retail outlets, restaurants, bars, and social settings such as parties—operate within the environment of the community.

Community strategies that focus on changing the local environment to decrease heavy drinking and reduce alcohol problems, among all age groups or specifically among young people, have the potential to effect structural changes in the community drinking environment that could have an especially broad and long-lasting impact on drinking behavior (see Holder 1997; Holder et al. 1997; Babor et al. 2003).

Research indicates that the prevention strategies most effective with minors and young adults are policy strategies that influence the price, availability, drinking context, or perceived risks of heavy drinking (Babor et al. 2003). Substantial changes in the conditions of sale (such as changing which outlets can legally sell alcohol and when they can do so) may alter young people’s access to alcohol as well as stimulate or reduce heavy drinking in this age group (Wagenaar et al. 1996). Similarly, introducing or legalizing specific beverage types (e.g., wine coolers, high-alcohol beer) appears to change beverage preferences and may increase alcohol consumption (see summary in Babor et al. 2003).

Federal as well as State laws—including those governing legal drinking age, licensing of alcohol outlets, the legal blood alcohol level for drinking and driving, service to obviously intoxicated people, and alcohol advertising—often form the basis for local policies. Local governments, in turn, are responsible for implementing and enforcing these laws. Examples of local government action can include giving priority to drinking-and-driving enforcement; mandating server training for bars, pubs, and restaurants; defining responsible alcoholic beverage service by licensed retail establishments; and allocating enforcement resources to prevent alcohol sales to people who are underage or obviously intoxicated. The relative emphasis that local police departments give to different alcohol-related policies is an example of the kind of administrative decision that is made locally.

To be effective, community prevention interventions require a mix of evidence-based program components and policy strategies. The National Institute on Alcohol Abuse and Alcoholism (NIAAA) has been at the forefront of encouraging evidence-based community prevention. Although many such efforts have been sponsored by other Federal and State agencies, this article discusses three research-based community prevention trials sponsored by NIAAA. These trial programs are comprehensive local efforts that use a combination of environmental strategies in concert to affect heavy drinking and related problems among all age groups, but especially among youth and young adults.

## The Saving Lives Project

The Saving Lives project was designed to reduce alcohol-impaired driving and related problems such as speeding (Hingson et al. 1996) in six communities in Massachusetts over a 5-year period. In each community a full-time city employee organized a task force of representatives of city departments to work on the project, which was funded at the rate of $1 per inhabitant annually to pay for the local coordinator, police enforcement, program activities, and educational materials. The task force designed the specific activities its community would implement. These included media campaigns, speeding and drunk-driving awareness days, telephone hotlines for reporting speeders, police training, high school peer-led education, establishment of Students Against Drunk Driving chapters, programs for college students, and information for retail alcohol outlets about drinking and risks.

Over the 5 years of the program, the participating communities saw a 25-percent reduction in fatal car crashes and more than a 40-percent reduction in alcohol-related fatal crashes relative to the rest of the State. The program effect was most pronounced among drivers between ages 15 and 25; among young adults in this age range there was a 39-percent reduction in fatal crashes compared with the rest of the State. In addition, program communities experienced a 5-percent reduction in crashes involving injuries that required medical attention and an 8-percent reduction in crash injuries among 16- to 25-year-olds.

The program did not significantly affect adults’ perceptions that police would stop drunk drivers and speeders. However, there were statistically significant increases in the number of 16- to 19-year-olds who believed that their licenses would be suspended if they were caught drinking and driving and that speeders would be stopped by police and fined substantially. In addition, 16- to 19-year-olds were half as likely to report driving after drinking in program communities, and there were 50 percent fewer citizen reports of speeding.

## The Community Trials Project

The Community Trials Project tested a five-component community intervention to reduce alcohol-related harm among people of all ages (see the figure), although epidemiological research has shown that young adult drinkers had higher-than-average risks of alcohol-related trauma (Holder et al. 1997). For this 5-year study conducted in California and South Carolina, three experimental and three matched comparison communities were selected, each with a population of approximately 100,000. Each community was racially diverse, 40 percent or more of its population being minority group members.

This project included five intervention components that were based on research about drinking patterns, risk, and sources of alcohol: (1) a Media and Mobilization component to develop community organization and support for the goals and strategies of the project, in part by using local news media; (2) a Responsible Beverage Service component to reduce service to intoxicated patrons at bars and restaurants; (3) a Sales to Youth component to reduce underage access; (4) a Drinking and Driving component to increase local enforcement of laws against driving while intoxicated; and (5) an Access component to reduce the availability of alcohol by affecting the number, location, and concentration of alcohol outlets.

Each community was to implement the basic minimum elements for each component. A local community coordinator worked with the research team, and each community was free to seek the best means and timing for implementing each component (see Treno and Holder 1997).

**Figure:**
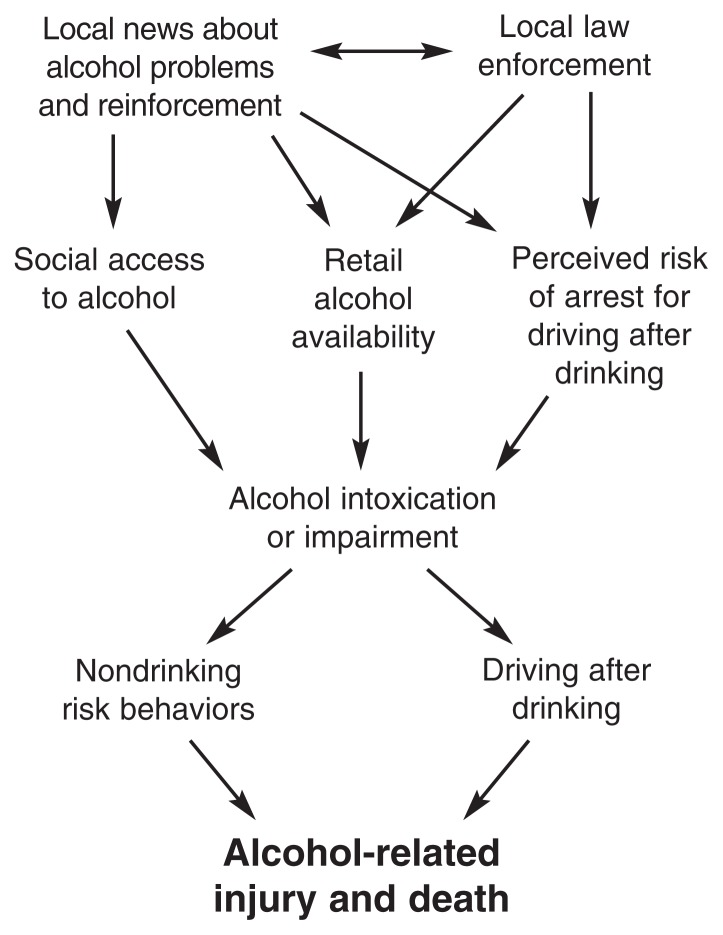
The Community Trials Project used several intervention components to reduce drinking-related harm among young adults. Local law enforcement and media coverage of alcohol problems and enforcement influenced retail alcohol availability, social access to alcohol, and perceived risk of arrest for drinking and driving. These intervention elements acted together to reduce alcohol-related injury and death.

Compared with control communities, communities in the intervention group experienced a 10-percent reduction in nighttime injury crashes and a 6-percent reduction in crashes in which police recorded that the driver had been drinking. Assault injuries seen in emergency departments in the intervention communities declined 43 percent compared with the rate seen in the comparison communities, and assault injuries requiring hospitalization declined by 2 percent, a statistically significant drop. Reports of driving after having had too much to drink declined 49 percent, and self-reports of driving when over the legal limit fell 51 percent. Surprisingly, although the size of the drinking population increased slightly in the experimental sites over the course of the study, there was a significant reduction in problematic alcohol use: The average number of drinks per occasion declined by 6 percent, and the variance in the frequency and volume of alcohol consumption (an indirect measure of heavy drinking) declined 21 percent (Holder et al. 2000).

Of particular interest for this article, the Sales to Youth component produced a significant reduction in alcohol sales to minors. This component consisted of training clerks and managers to conduct age identification checks, implementing effective policies governing licensed alcohol stores, and especially, threatening legal sanctions against alcohol outlets that sell to minors (Grube 1997). Overall, alcohol retailers in experimental communities were half as likely as retailers in control communities to sell alcohol to minors.

## Communities Mobilizing for Change on Alcohol

Communities Mobilizing for Change on Alcohol (CMCA) was a community-organizing effort to reduce underage access to alcohol by changing local policies and practices (Wagenaar et al. 1994). Fifteen communities in Minnesota and western Wisconsin were matched and randomly assigned to the intervention or control condition, resulting in seven intervention sites and eight comparison sites, ranging in population from 8,000 to 65,000. Specific prevention activities varied across communities.

Each experimental community, with the assistance of a local coordinator, was free to develop an approach to curtailing underage drinking by reducing alcohol availability to underage drinkers. In all cases, communities were encouraged to use alcohol policy strategies that emphasized changes in the local drinking and alcohol sales environment.

After the fifth year of the project, the intervention communities, compared with control communities, reported more awareness of the need to regulate alcohol sales to youth (Wagenaar et al. 1996). Surveys revealed that merchants checked for age identification more often and made fewer sales to minors, results which were confirmed by compliance checks using young-looking alcohol purchasers. Alcohol sales to minors decreased by 10.2 percent for restaurants and bars and 4.57 percent for liquor stores. A telephone survey indicated that 18- to 20-year-olds in the intervention communities were less likely than those in the control communities to consume alcohol themselves and less likely to provide it to others who were underage (Wagenaar et al. 2000*a*). The interventions reduced both drinking and drinking-related behavior (i.e., driving after drinking, attempting to purchase alcohol, and providing alcohol to minors) among 18- to 20-year-olds; that is, 7 percent fewer young people reported drinking during a 30-day period, and the number of drinking occasions declined 4 percent. Compared with the control communities, the intervention communities saw fewer drinking-and-driving arrests and fewer disorderly conduct violations among 15- to 17-year-olds (Wagenaar et al. 2000*b*).

## Learning From Local Efforts

The three NIAAA-sponsored community prevention projects described provide strong evidence of the positive effects of research-based local prevention efforts that take a comprehensive approach using a combination of strategies. Studies by other researchers have demonstrated that comprehensive strategies can effect substantial changes in alcohol-related behavior (Clapp et al. 2005; Hingson et al. 2005; Lewis et al. 1996; Preusser et al. 1994; Weitzman et al. 2004).

For example, Clapp and colleagues (2005) studied a yearlong intervention designed to prevent driving under the influence (DUI) on a university campus. The intervention consisted of increased law enforcement (DUI checkpoints and roving DUI patrols) combined with extensive media advocacy (placement of stories on local television news broadcasts and in the campus newspaper) and a student-designed advertising campaign, both intended to heighten awareness of the increased enforcement. The researchers found that self-reported instances of DUI decreased significantly at the intervention school; DUI rates at a comparison university remained stable.

Recently, Hingson and colleagues (2005) examined alcohol-related traffic fatality data from a subset of communities that were awarded substance abuse prevention grants through the Robert Wood Johnson Foundation’s Fighting Back program. Investigators selected five communities that implemented a concentrated communitywide effort to restrict alcohol availability and expand treatment services. Comparing rates of fatal traffic crashes during the 10 years of the study with rates during the 10-year period before the study, the researchers found that alcohol-related fatal crashes declined significantly during the years of the program in both intervention and comparison communities. Overall, the declines ranged from 17 percent to 22 percent, depending on the blood alcohol concentration (BAC) associated with the crash. In the three communities in which the intervention targeted the entire city, alcohol-related fatal crashes declined from 31 percent to 39 percent, depending on BAC. Communities with less comprehensive intervention programs did not experience such improvements relative to their comparison communities.

These projects’ findings confirm that local policies to reduce young adult drinking or alcohol-related problems are most likely to be effective when they are adequately enforced and when the intended targets of the intervention are aware of both the policies and their enforcement.

Community action projects are intended to address the total community system and are not naturally limited to a specific target group or service group. To reduce alcohol problems in the entire community, local leadership is required in designing, implementing, and supporting new alcohol policies. To ensure that the strategies were research based, the research teams for the Saving Lives, Community Trials, and CMCA projects designed (or identified) prevention strategies that research had shown to be effective and supported (or assisted) the communities in implementing those activities and policies. Communities took responsibility for determining how best to implement each strategy.

Although local alcohol policy strategies have the best chances for success when they draw on scientific evidence, many community prevention projects involve interventions that research has indicated are unlikely to reduce alcohol-related problems. For example, many communities pursue public education efforts designed to produce attitude change. Such educational strategies often are focused on information alone, based on the assumption that an informed community will necessarily experience a reduction in alcohol-related problems. However, there is no evidence that education alone can reduce alcohol-related problems at the community level (see Babor et al. 2003 for a recent review of the evidence concerning the effectiveness of educational programs and prevention).

Enforcement of underage drinking laws can be significantly enhanced with modest increases in community support (e.g., when elected officials publicly endorse the effort and work to implement the policies, or when community residents give public support via letters to the editor). Even moderate increases in enforcement can reduce outlet sales of alcohol to minors, especially when combined with media campaigns and other community and policy activities (Grube 1997; Wagenaar et al. 2000*a*).

Communication with the entire community is essential. The most effective and inexpensive way to increase public support for strategies that reduce alcohol availability is to use local newspapers, radio, and television—a strategy sometimes called media advocacy (Casswell 1995; Holder and Treno 1997; Treno and Holder 1997; Wallack 1990). Without skillful media work, it is very difficult, and perhaps impossible, to implement policy-driven structural changes within a community because such strategies depend upon the support and leadership of the community to effectively implement and reinforce the effort. Media advocacy can be used to make retailers, underage buyers, parents, and other adults more aware of the likelihood of legal consequences for selling or providing alcohol to people who are underage, and to enhance community perceptions of ownership of intervention strategies.

The long-term test of successful community prevention is whether local governments continue to implement the policies after the project ends, and whether the policies themselves continue to be effective. Once implemented, local policies often can have a longer life than services that must be maintained and funded each year. Because policies typically affect communities’ administrative codes and procedures (e.g., local zoning and land use regulations governing alcohol retailers), their maintenance does not necessarily require additional funds. Thus, for example, a local policy requiring training for alcohol beverage servers in bars and restaurants, to be provided by an existing adult education system, could be effective for a longer period than a professionally planned public education campaign that must be renewed annually.

Even when a strategy becomes less effective over time—as compliance, regulation, or enforcement declines—it can continue to have a sustained effect even without reinforcement. For example, enforcing bans on selling alcohol to minors increases retailers’ compliance with the law. But even if enforcement drops off, there may be residual compliance. A second illustration concerns drinking-and-driving trends after the minimum legal drinking age was raised to age 21 for all U.S. States. O’Malley and Wagenaar (1991) found that the incidence of drinking and driving declined when the drinking age was raised to 21, despite varied and often low levels of enforcement. Further, young people in States with a higher minimum drinking age actually sustained lower average drinking levels even after they reached the legal drinking age and were no longer prohibited from drinking.

In the final analysis, complementary strategies such as those described above, where local government implements and enforces alcohol-related policies aimed at restructuring the community’s total alcohol environment, are more likely to be effective in achieving their goals than single-intervention strategies that are only in place for the duration of a special project.
